# Evaluation of a new human immunodeficiency virus antigen and antibody test using light-initiated chemiluminescent assay

**DOI:** 10.3389/fcimb.2025.1474127

**Published:** 2025-01-31

**Authors:** Yijun Li, Fangfang Jin, Yunhui Li, Yan Li, Yajie Wang, Ximing Yang

**Affiliations:** ^1^ Clinical Laboratory, Dongzhimen Hospital, Beijing University of Chinese Medicine, Beijing, China; ^2^ Clinical Laboratory, Beijing Ditan Hospital, Capital Medical University, Beijing, China

**Keywords:** human immunodeficiency virus, light-initiated chemiluminescent assay, homogeneous immunoassay, performance evaluation, LiCA^®^

## Abstract

**Objectives:**

The goal of this study is to evaluate the analytical and clinical performance of a new human immunodeficiency virus antigen and antibody (HIV Ag/Ab) test using light-initiated chemiluminescent assay (LiCA^®^) and compare it with the well-established Architect^®^ HIV Ag/Ab combo assay in a clinical setting.

**Methods:**

We used banked samples and national reference controls to identify the ability to detect HIV Ag/Ab and different viral subtypes. Thirteen seroconversion panels were tested to evaluate early detection of HIV. A total of 21,042 patient samples were collected to compare the diagnostic performance of LiCA^®^ with Architect^®^. Screening-reactive results were confirmed by Western blotting and nucleic acid testing.

**Results:**

Total imprecision was within 2.49%–6.56%. The C_5_–C_95_ interval was within −10.20%–7.67% away from C_50_. The limit of detection for p24 antigen was <1.00 IU/mL. Using national reference panels and banked sample pools, LiCA^®^ successfully detected all negative and positive controls in line with the criteria, and all HIV-positive specimens containing different viral subtypes. In 13 seroconversion panels, LiCA^®^ detected reactive results on average 5.73 days (95% CI: 3.42–8.04) after the initial RNA test results were confirmed positive, which was 1.27 days earlier (−3.75 to 1.21) compared to Architect^®^. Paired comparisons in 21,042 clinical patient samples demonstrated that LiCA^®^ detected HIV Ag/Ab with a slightly better performance in sensitivity (100.00% vs. 99.65%), specificity (99.85% vs. 99.81%), negative predictive value (NPV, 100.00% vs. 99.99%), and positive predictive value (PPV, 89.84% vs. 87.85%) than Architect^®^. Total agreement between two assays was 99.67% with a kappa value of 0.89.

**Conclusion:**

LiCA^®^ HIV Ag/Ab is a precise and highly sensitive assay for measuring HIV-1 p24 antigen and HIV-1/2 antibodies with differentiated S/Co values of Ag/Ab. The assay is appropriate for use in the clinical routine test for the early detection of HIV.

## 1 Introduction

Acquired immune deficiency syndrome (AIDS), caused by the human immunodeficiency virus (HIV), is a global infectious disease that seriously endangers human health. HIV attacks the patient’s immune system, which causes a variety of complications and even death. In addition, the virus continues to be spread through blood transfusions, sexual contact, and drug use (Volberding and Deeks, 2010). As reported by the US Centers for Disease Control and Prevention (CDC), approximately 1.7 million people were newly infected with HIV worldwide in 2018, and 770,000 people died among those living with AIDS (UNAIDS, 2019). Of added concern is that several African countries and Middle East nations are far from controlling this epidemic (El-Sadr et al., 2019). Fortunately, continued access to antiretroviral therapy (ART) in the early stages of infection can have a major positive effect on reducing transmission and death among those living with HIV (Cohen et al., 2011; Rodger et al., 2019). Therefore, early diagnosis of the infection and linking the patients to the proper medicinal therapy are critical for the management of AIDS patients. This heavily relies on an effective viral screening strategy.

During recent decades, various methods for detecting HIV antibody (Ab), p24 antigen (Ag), and ribonucleic acid (RNA) in serum or plasma have been developed for the diagnosis of HIV infection (Alexander, 2016; Gray et al., 2018). The detection capabilities of various methods exhibit certain variations. For instance, the sensitivity of first-generation HIV-Ab reagents is 99%, with a specificity ranging from 95% to 98% (Alexander, 2016). Taking the Alere HIV Combo POCT test as an example for HIV-1 P24 antigen detection, its sensitivity is 88%, and its specificity is 100% (Fitzgerald et al., 2017). The detection capability of HIV RNA is primarily reflected in its sensitivity. Currently, the lower limit of detection for ordinary HIV RNA quantitative reagents is 100–200 copies/mL, while high-sensitivity quantitative reagents can achieve a lower limit of detection as low as 20 copies/mL. In general, a suspected subject is initially detected with the screening test for HIV Ag/Ab, and the screening-reactive assay is further confirmed by Western blot (WB) and/or nucleic acid test (NAT) to clarify the infection condition or even a false-positive result (Centers for Disease Control and Prevention, 2018; Branson, 2019). The Ag/Ab screening test with higher sensitivity and specificity can be more favorable in clinical practice due to its better detection capability for viral infection from first- to fifth-generation commercial kits (Alexander, 2016). Unlike the third-generation assay that detects Ab alone, the fourth- and fifth-generation assays detect both Ab and p24 Ag in combination, reducing the test-negative window to 8–14 days (Alexander, 2016; Qiu et al., 2017). Moreover, the fifth-generation assay can differentiate Ab and Ag reactivity instead of a single result by the fourth-generation kit, thus facilitating the confirmatory strategy for early detection of HIV (Muhlbacher et al., 2019; Yang et al., 2022).

Here, we introduce a new fifth-generation HIV Ag/Ab combination test that is based on the light-initiated chemiluminescent assay (LiCA^®^) (Bian et al., 2018). LiCA^®^ provides a fully automatic homogeneous immunoassay platform, which has been widely developed for detection of various analytes with high sensitivity and specificity, such as hormones (Yu et al., 2021; Wang et al., 2022), cardiac proteins (Yang et al., 2022; Li et al., 2024), and Ag and Ab (Li et al., 2023; Yu et al., 2023). In this study, we aim to evaluate the performance of the LiCA^®^ HIV Ag/Ab assay in analytical and clinical perspectives and compare it with the well-established Architect^®^ HIV Ag/Ab combo test in clinical setting.

## 2 Materials and methods

### 2.1 Sample collection and HIV Ag/Ab serological assays

**Figure 1:**
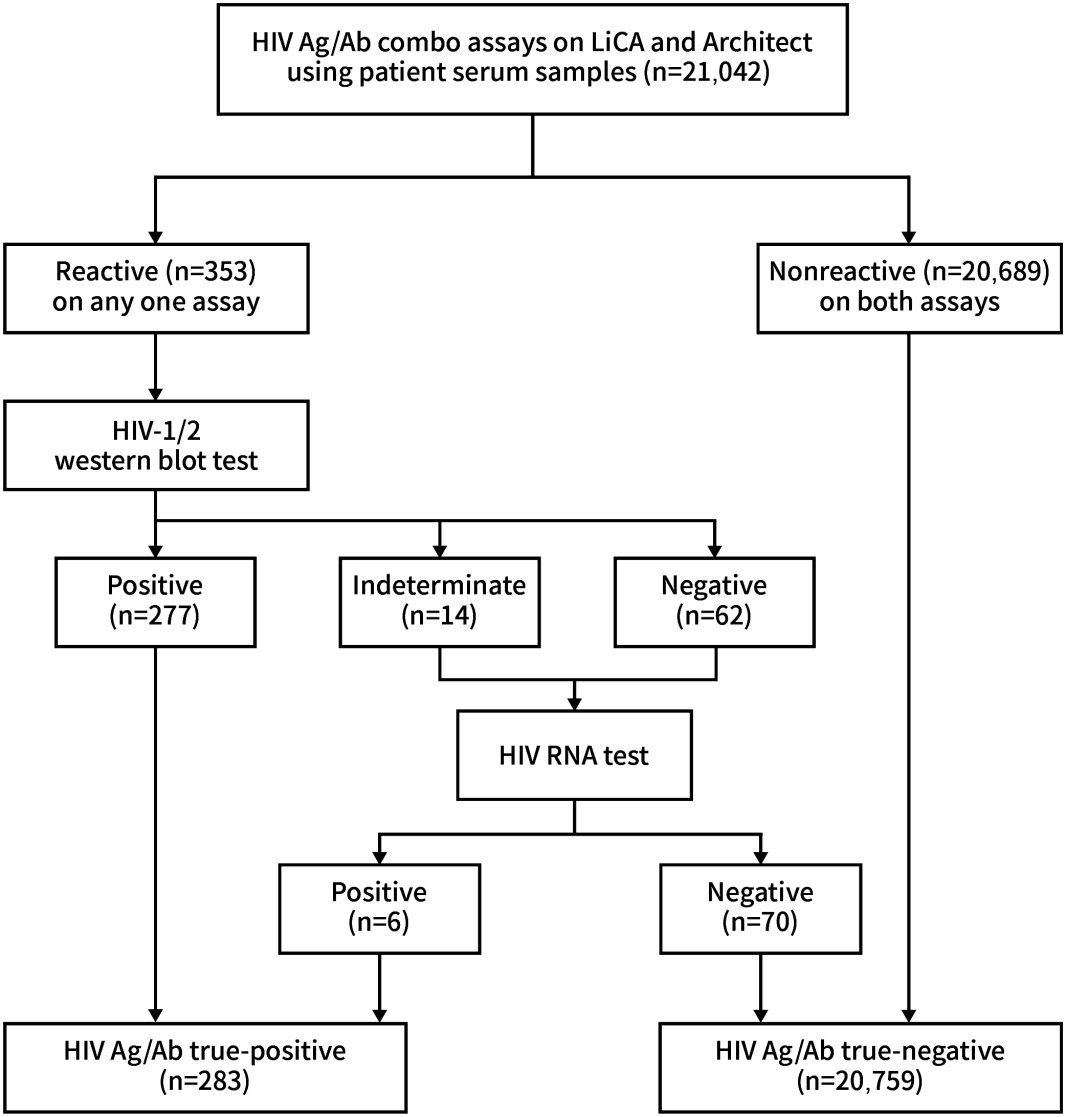
HIVAg/Ab combo assays on LiCA and Architectusing patient serum samples.

**Figure 2:**
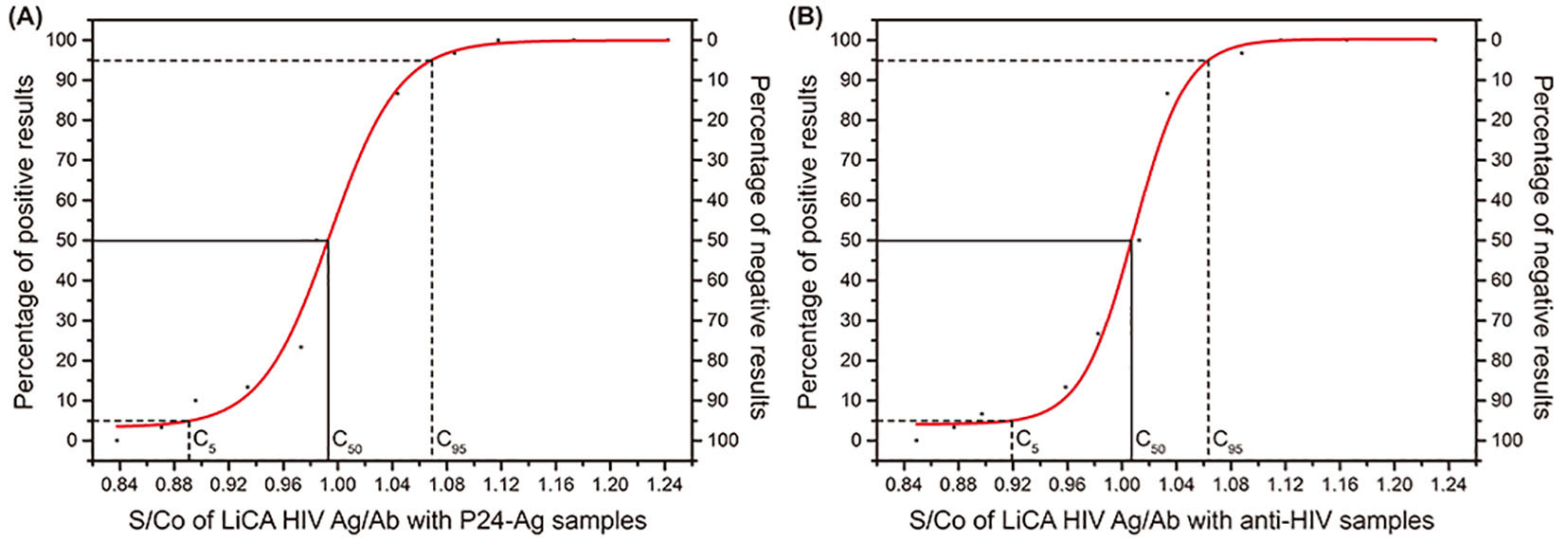
The S/Co of LiCA HIV Ag/Ab with P24-Ag samples and anti-HlV samples. **(A)** S/Co of LiCA HIV Ag/Ab with P24-Ag samples. **(B)** S/Co of LiCA HIV Ag/Ab with anti-HlV samples.

We recruited a total of 21,042 clinical serum specimens from inpatients and outpatients in Dongzhimen Hospital. HIV Ag/Ab screening tests were performed on the LiCA^®^ 500 platform (Chemclin Diagnostics, Beijing, China) and the Architect^®^ i2000SR system (Abbott Laboratories, IL, USA) in parallel (**Figure 1**). LiCA^®^ HIV Ag/Ab is a one-step fully automatic homogeneous immunoassay. Serum samples were dispensed into two cuvettes for detecting antibodies to HIV-1/HIV-2 subtypes and HIV-1 p24 antigen, respectively. The Ag/Ab reactivity can be differentiated in results. A signal-to-cutoff (S/Co) ratio ≥1.0 in any cuvette is considered to be screening-reactive. Time to the first report is approximately 25 min. Architect^®^ HIV Ag/Ab combo is a two-step indirect immunoassay and reports combined Ag/Ab reactivity in a single result. A ratio of S/Co ≥1.0 is regarded as screening-reactive. Any one assay with a reactive S/Co was retested in duplicate. The repeated screening-reactive assays were then allocated for antibody identification with the WB test of *recom*Line HIV-1/HIV-2 IgG (Mikrogen Diagnostics, Neuried, Germany). Subjects with WB-indeterminate and WB-negative results were further identified with the Cobas^®^ AmpliPrep/Cobas^®^ TagMan^®^ HIV-1 RNA test (Roche Diagnostics, Mannheim, Germany). Finally, the HIV Ag/Ab true-positive group included both WB-positive and RNA-positive results, and the true-negative group included those with screening-negative results in both assays and RNA-negative results. The testing protocol was plotted as shown in **Figure 2** (Alexander, 2016; Fitzgerald et al., 2017).

### 2.2 Precision study

We performed precision analysis in S/Co ratios according to the EP15-A3 protocol of the Clinical and Laboratory Standards Institute (CLSI) (CLSI, 2014), using three levels of patient serum samples, and two levels of controls for HIV antibodies and p24 antigen, respectively. An acceptable coefficient of variation (CV) was ≤15%. In addition, we followed the guideline of EP12-A2 (CLSI, 2008) and prepared a series of dilutions with a positive sera to determine the C_50_ target and the C_5_–C_95_ interval for HIV antibodies and p24 antigen, respectively. An acceptable C_5_–C_95_ interval was within C_50_ ± 15%.

### 2.3 Detection capability

We evaluated the assay detection capability to HIV antibodies and p24 antigen with the reference panels of the China National Institutes for Food and Drug Control (NIFDC). The panel for HIV-1 p24 antigen (Lot 220015-201906) was composed of 20 negative controls, 10 positive controls, and 10 levels of serial dilutions for study of the limit of detection (LoD). The panel for HIV antibodies (Lot 370045-201901) contained different types of negative and positive controls for the detection of HIV-1/HIV-2 subtypes and controls for the LoD study of B/B′, BC, and AE genotypes. Furthermore, we prepared a series of doubling dilutions to quantify the assay LoD to HIV-1 p24 antigen using the NIFDC reference panel (Lot 220015-201906, baseline p24 concentration 20 IU/mL) and the World Health Organization (WHO) international standard from the National Institute for Biological Standards and Control (NIBSC, code 90/636, baseline p24 concentration 1,000 IU/mL).

### 2.4 Detection of seroconversion panels and HIV subtypes

We used 74 banked clinical samples with identified HIV subtypes, 79 HIV-positive patient sera with a low level of S/Co in 1.0–35.0, and 13 commercial seroconversion panels for comparative detection between LiCA^®^ and Architect^®^ HIV Ag/Ab assays. Seroconversion panels were purchased from BioMex (*n* = 2, SCP-HIV 005–006, Heidelberg, Germany), ZeptoMetrix (*n* = 6, PIHIV 9011–9077, Franklin, MA, USA), and SeraCare (*n* = 5, PRB 953–977, Milford, MA, USA).

### 2.5 Potential interference and cross-reactivity

To evaluate potential interferences from bilirubin, triglycerides, hemoglobin, and biotin, two patient sera (baseline S/Co 0.72 and 5.68) were used as the diluent to prepare a pool of samples with a serial concentration of each interferent, respectively. The cross-reactivity study was performed using 169 serum specimens free of HIV but positive for potential interferents, such as auto-antibodies and other viral infection. All samples were tested in duplicate. A significant interference was considered when the recovery change of mean S/Co was ≥15% in the sample with a baseline S/Co >0.8 or a reactive result was recorded in the sample with a baseline S/Co <0.8.

### 2.6 Statistics

Statistical analyses were conducted using MedCalc (MedCalc Software, Mariakerke, Belgium) and Excel (Microsoft, WA, USA). Agreement between LiCA^®^ and Architect^®^ was analyzed based on the screening-reactive (S/Co ≥1.0) or -nonreactive (S/Co <1.0) assay results. Specifically, using the HIV Ag/Ab test kit, HIV-negative samples, HIV p24 antigen-positive samples, and HIV antibody-positive samples were tested. Through the ROC curve, with the maximum Youden index as the criterion, the optimal cutoff signal values corresponding to HIV p24 antigen and HIV antibody were obtained, respectively. The ratio of the sample detection signal value to the cutoff signal value was defined as S/Co. Since the signal value corresponding to CO represents the optimal threshold for distinguishing between negative and positive results, the S/Co value of 1 was used as the cutoff for this distinction.

Diagnostic performance parameters, such as sensitivity, specificity, negative predictive value (NPV), and positive predictive value (PPV) were determined with confirmatory results by WB and RNA tests. The *t*-test was used to evaluate the significant difference between paired samples. *p*-value <0.05 was considered as statistically significant.

## 3 Results

### 3.1 Precision analysis

A precision study on S/Co ratios presented that the assay CVs for repeatability and within-lab imprecision were 2.49%–5.11% on patient sera, 2.93%–5.05% on p24 antigen controls, and 3.73%–6.56% on anti-HIV controls (**Table 1**). The C_50_ target and the C_5_–C_95_ interval away from C_50_ were 0.99 S/Co and −10.20%–7.67% for p24 antigen and 1.01 S/Co and −8.79%–5.64% for HIV antibodies, respectively (**Supplementary Figure 1**).

**Table 1:** Precision study for the LiCA^®^ HIV Ag/Ab assay.

Sample	Mean	Repeatability		Within-lab imprecision	
	(S/Co^a^)	SD^a^	% CV^a^	SD	% CV
Serum 1	2.63	0.07	2.49	0.10	3.95
Serum 2	11.55	0.37	3.19	0.53	4.60
Serum 3	37.61	1.18	3.15	1.92	5.11
p24 antigen QC1	1.56	0.05	3.52	0.08	5.05
p24 antigen QC2	3.20	0.09	2.93	0.14	4.47
Anti-HIV QC1	1.14	0.04	3.73	0.07	6.56
Anti-HIV QC2	2.38	0.09	3.88	0.15	6.46

a S/Co, signal-to-cutoff ratio; SD, standard deviation; CV, coefficient of variation.

### 3.2 Detection capability

Using China national reference control panels (**Table 2**), LiCA^®^ presented nonreactive results in all negative controls for both HIV-1 p24 antigen and HIV antibodies. The mean S/Co ratios with 95% confidence interval (95% CI) were 0.33 (0.31–0.34) for p24 and 0.23 (0.19–0.27) for antibodies, respectively. All positive controls for p24 were detected to be reactive with a mean S/Co of 31.45 (19.03–43.89). One positive control for the antibody of HIV-1 group O (*n* = 3) was undetected (S/Co = 0.32). All other positive controls for HIV antibodies were measured with reactive S/Co ratios at a mean of 55.30 (22.84–87.76). The LoD for p24 antigen was identified to be ≤1.25 IU/mL. All positive controls for LoDs to B/B′, BC, and AE genotypes were recorded with reactive S/Co values at a mean of 11.66 (4.80–18.53).

**Table 2:** Detection of the national reference panels for the LiCA^®^ HIV Ag/Ab assay.

Sample types	Sample no.	*n*	Acceptable criteria	Test results	S/Co^a^ mean (95% CI^a^)
National reference panel for HIV-1 p24 antigen					
Negative control	N1–N20	20	Nonreactive results = 20/20	Nonreactive results = 20/20	0.33 (0.31–0.34)
Positive control	P1–P10	10	Reactive results = 10/10	Reactive results = 10/10	31.45 (19.03–43.89)
LoD^a^ control	L1–L10	10	L10 = nonreactive, and LoD ≤2.50 IU/mL	L10 = nonreactive, and LoD ≤1.25 IU/mL	Not applicable
National reference panel for HIV antibodies					
Negative control	N1–N13	13	Nonreactive results = 13/13	Nonreactive results = 13/13	0.23 (0.19–0.27)
Positive control for HIV-1 group M subtype	P1–P14	14	Reactive results = 14/14	Reactive results = 14/14	72.45 (31.35–113.55)
Positive control for HIV-1 group O subtype	P15–P17	3	Reactive results ≥1/3	Reactive results = 2/3	4.40 (0.32–11.48)
Positive control for HIV-2 subtype	P18–P20	3	Reactive results ≥2/3	Reactive results = 3/3	7.83 (2.07–13.03)
LoD control for B/B′ genotype	BB1–BB5	5	Reactive results ≥3/5	Reactive results = 5/5	19.02 (5.17–35.61)
LoD control for BC recombinant genotype	BC1–BC5	5	Reactive results ≥3/5	Reactive results = 5/5	10.13 (2.63–19.75)
LoD control for AE recombinant genotype	AE1–AE5	5	Reactive results ≥3/5	Reactive results = 5/5	5.84 (1.78–10.95)

a S/Co, signal-to-cutoff ratio; 95% CI, 95% confidence interval; LoD, limit of detection.

To further clarify the assay LoD to p24 antigen, linear regression analyses were performed between the low range (0–10 IU/mL) of p24 antigen concentrations (*Y*) and assay S/Co values (*X*). For the NIFDC reference material, the regression equation was *Y* = 1.091*X* − 0.365 (*R* = 0.999) and LoD was calculated to be 0.73 IU/mL. For the WHO international standard, the equation was *Y* = 2.908*X* − 2.008 (*R* = 0.999) and LoD was assessed to be 0.90 IU/mL (**Supplementary Table 1**).

### 3.3 Detection of seroconversion panels and HIV subtypes

Thirteen seroconversion panels were measured to evaluate early detection of HIV (**Table 3**). Among them, LiCA^®^ presented 2/13 with earlier, 1/13 with later, and 10/13 with equal detections in comparison to Architect^®^. In general, LiCA^®^ detected the panels with an average of 5.73 (95% CI, 3.42–8.04) days at the first reactive result since the RNA-positive detection, which had a mean of −1.27 (−3.75 to 1.21) days earlier than Architect^®^. The relative sensitivity coefficient was −0.08 (−0.38 to 0.22).

**Table 3:** Detection of seroconversion panels.

Panel no. (*n* = 13)	Sample size	Numbers of nonreactive bleeds			Days at the first reactive result since RNA (+)		
		LiCA^®^	Architect^®^	LiCA^®^ vs. Architect^®^	LiCA^®^	Architect^®^	LiCA^®^ vs. Architect^®^
SCP-HIV-005	25	4	4	0	/	/	/
SCP-HIV-006	17	2	2	0	/	/	/
PIHIV9011	11	8	9	−1	0	8	−8
PIHIV9016	10	8	8	0	3	3	0
PIHIV9020	22	19	19	0	7	7	0
PIHIV9021	17	14	13	1	7	4	3
PIHIV9031	19	15	16	−1	6	15	−9
PIHIV9077	24	11	11	0	4	4	0
PRB953	4	2	2	0	7	7	0
PRB955	5	2	2	0	7	7	0
PRB971	4	2	2	0	7	7	0
PRB974	4	2	2	0	2	2	0
PRB977	4	2	2	0	13	13	0
Total	166	91	92	−1	63	77	−14
Mean	12.77	7.00	7.08	−0.08	5.73	7.00	−1.27
95% CI^a^	7.79–17.74	3.37–10.63	3.41–10.74	−0.38–0.22	3.42–8.04	4.31–9.68	−3.75–1.21

a 95% CI, 95% confidence interval.

Detection of various HIV subtypes in clinical patient sera was evaluated with 64 specimens containing HIV antibodies to different types of HIV subtypes, 10 p24 antigen single positive samples, and 79 weak positive cases that were collected from HIV-confirmed patients and measured with low reactive S/Co values (1.0–35.0) by the Architect^®^ HIV Ag/Ab combo assay. Both LiCA^®^ and Architect^®^ successfully recorded reactive results for all subjects studied (**Table 4**).

**Table 4:** Detection of different types of HIV-positive serum samples.

Sample types	Sample size	Reactive samples on LiCA^®^		Reactive samples on Architect^®^	
		*n*	S/Co^a^ mean (95% CI^a^)	n	S/Co mean (95% CI)
B/B′ genotype	10	10	321.62 (213.29–429.95)	10	547.62 (275.39–819.85)
BC recombinant genotype	13	13	457.98 (304.51–611.46)	13	411.28 (251.01–571.56)
AE recombinant genotype	38	38	372.83 (326.32–419.34)	38	269.91 (203.06–336.76)
HIV-2 subtype	2	2	156.30 (106.44–206.24)	2	535.45 (16.34–1,054.62)
HIV-1 group O subtype	1	1	20.09 (20.09–20.09)	1	6.70 (6.70–6.70)
HIV-1 p24 antigen single positive	10	10	21.81 (13.68–29.94)	10	30.85 (14.87–46.83)
Weak positive^*^	79	79	72.19 (60.90–85.49)	79	13.97 (10.05–17.92)
Total	153	153	189.23 (158.52–219.95)	153	153.45 (113.63–193.26)

a S/Co, signal-to-cutoff ratio; 95% CI, 95% confidence interval.

^*^The weak positive specimens were collected from HIV-positive patients and measured with a low reactive signal-to-cutoff ratio between 1.0 and 35.0 by Architect^®^ HIV Ag/Ab combo.

### 3.4 Cross-reactivity and interference

The assay recovery changes were determined to be −4.01%–4.76%, −4.91%–4.74%, −2.62%–4.79%, and −2.15%–6.98% on LiCA^®^ by spiking potential interferents (up to 342.08 µmol/L bilirubin, 33.90 mmol/L triglycerides, 5.00 g/L hemoglobin, and 102.25 nmol/L biotin) into both low and high levels of specimens (baseline S/Co 0.72 and 5.68), respectively. In all 169 samples free of HIV but positive for potential interfering factors such as auto-antibodies, other viral infections, and multiple pregnancies, no test-reactive results were observed on both LiCA^®^ and Architect^®^ (**Supplementary Table 2**).

### 3.5 Comparison of diagnostic performance between LiCA^®^ and Architect^®^

Among 21,042 clinical patient sera recruited, 283 (1.34%) were confirmed to be HIV-positive and 20,759 (98.66%) were HIV-negative (**Figure 2**). Compared to Architect^®^, LiCA^®^ presented a slightly better but not significantly different (*p* > 0.05) performance in sensitivity (100.00% vs. 99.65%), specificity (99.85% vs. 99.81%), NPV (100.00% vs. 99.99%), PPV (89.84% vs. 87.85%), and overall accuracy (99.85% vs. 99.81%) for the diagnosis of HIV infection (**Table 5**).

**Table 5:** Assay performance in patient serum samples (95% confidence interval).

*n* = 21,042	LiCA^®^	Architect^®^
Sensitivity, %	100.00% (98.71–100.00%)	99.65% (98.05–99.99%)
Specificity, %	99.85% (99.78–99.90%)	99.81% (99.74–99.87%)
Negative predictive value, %	100.00% (98.71–100.00%)	99.99% (99.96–99.99%)
Positive predictive value, %	89.84% (86.22–92.59%)	87.85% (84.09–90.82%)
Accuracy, %	99.85% (99.79–99.90%)	99.81% (99.74–99.86%)

With further analysis of the segmented S/Co values (**Table 6**), we found that LiCA^®^ detected true-positive results with a portion of 10.34% (*n* = 29), 60.00% (*n* = 10), 88.89% (*n* = 9), 94.74% (*n* = 19) and 100% (*n* = 248), and 89.84% (*n* = 315) in an S/Co range of 1.00–4.99, 5.00–9.99, 10.00–29.99, 50.00–99.99 and ≥100.00, and in overall reactive S/Co ratios (≥1.00), respectively. In contrast, the corresponding true-positive detection portions on Architect^®^ were 7.41% (*n* = 32), 44.44% (*n* = 9), 89.47% (*n* = 19), 94.29% (*n* = 35) and 100% (*n* = 226), and 87.85% (*n* = 321), respectively. One case, which was confirmed to be HIV-positive, was misdetected on Architect^®^ (S/Co = 0.59) but strongly reactive on LiCA^®^ (S/Co = 64.14).

**Table 6:** Comparisons between LiCA^®^ and Architect^®^ HIV Ag/Ab assays in patient serum samples (*n* = 21,042).

S/Co^a^ segmentation	LiCA^®^			Architect^®^			LiCA^®^ vs. Architect^®^
	No. (%) of samples	False-negative or true-positive	True-negative or false-positive	No. (%) of samples	False-negative or true-positive	True-negative or false-positive	Agreement (95% CI^a^)
<1.00 (nonreactive)	20,727 (98.50%)	0 (0.00%)	20,727 (100.00%)	20,721 (98.47%)	1 (0.01%)	20,720 (99.99%)	99.85% (99.78–99.90%)
≥1.00 (reactive)	315 (1.50%)	283 (89.84%)	32 (10.16%)	321 (1.53%)	282 (87.85%)	39 (12.15%)	88.16% (84.11–91.49%)
1.00–4.99	29 (0.14%)	3 (10.34%)	26 (89.66%)	32 (0.15%)	2 (7.41%)	25 (92.59%)	9.38% (1.98–25.02%)
5.00–9.99	10 (0.05%)	6 (60.00%)	4 (40.00%)	9 (0.04%)	4 (44.44%)	5 (55.56%)	44.44% (13.70–78.80%)
10.00–29.99	9 (0.04%)	8 (88.89%)	1 (11.11%)	19 (0.09%)	17 (89.47%)	2 (10.53%)	89.47% (66.86–98.70%)
50.00–99.99	19 (0.09%)	18 (94.74%)	1 (5.26%)	35 (0.17%)	33 (94.29%)	2 (5.71%)	94.29% (80.84–99.30%)
≥100.00	248 (1.18%)	248 (100.00%)	0 (0.00%)	226 (1.07%)	226 (100.00%)	0 (0.00%)	100.00% (98.38–100.00%)
Total	21,042 (100.00%)	283 (1.34%)	20,759 (98.66%)	21,042 (100.00%)	283 (1.34%)	20,759 (98.66%)	99.67% (99.58–99.74%)

a S/Co, signal-to-cutoff; 95% CI, 95% confidence interval.

### 3.6 Agreement between LiCA^®^ and Architect^®^

Paired comparisons demonstrated that the overall agreement between LiCA^®^ and Architect^®^ was 99.67% (95% CI, 99.58–99.74%, *n* = 21,042) and the Cohen’s kappa was 0.89 (0.86–0.91). Agreements in nonreactive and reactive assays were 99.85% (99.78%–99.90%, *n* = 20,721) and 88.16% (84.11%–91.49%, *n* = 321), respectively (**Table 6**). The S/Co segmentation analysis for reactive results revealed that more detailed agreements in a range of 1.00–4.99, 5.00–9.99, 10.00–29.99, 50.00–99.99, and ≥100.00 were 9.38% (*n* = 32), 44.44% (*n* = 9), 89.47% (*n* = 19), 94.29% (*n* = 35), and 100% (*n* = 226), respectively. There were 70 (0.33%, *n* = 21,042) discrepant results between these two assays (**Table 7**). Architect^®^ contributed 38 (54.29%) false-positive subjects and 1 (1.43%) false-negative subject. The remaining 31 (44.28%) cases had false-positive reactivity on LiCA^®^. Among these 70 discrepancies, 63 (90.00%) were identified as having false-positive reactivity in a low range of S/Co values (<10.00) either from Architect^®^ (34/63, 53.97%) or from LiCA^®^ (29/63, 46.03%).

**Table 7:** Analysis of discrepant assays between LiCA^®^ and Architect^®^ HIV Ag/Ab combo in patient serum samples (*n* = 70).

S/Co segmentation	No. (%)^*^ of samples	LiCA^®^ false-positive	LiCA^®^ false-negative	Architect^®^ false-positive	Architect^®^ false-negative
1.00–4.99	54 (77.14%)	25 (46.30%)	0 (0.00%)	29 (53.70%)	0 (0.00%)
5.00–9.99	9 (12.86%)	4 (44.44%)	0 (0.00%)	5 (55.56%)	0 (0.00%)
≥10.00	7 (10.00%)	2 (28.57%)	0 (0.00%)	4 (57.14%)	1 (14.29%)
Total	70 (100.00%)	31 (44.28%)	0 (0.00%)	38 (54.29%)	1 (1.43%)

^*^The sample amount included subjects with a candidate segment of reactive signal-to-cutoff (S/Co) ratios either on LiCA^®^ or on Architect^®^ among the discrepant cohort.

## 4 Discussion

The screening test to HIV Ag/Ab is the initial step for the diagnosis of HIV infection (Centers for Disease Control and Prevention, 2018; Branson, 2019). The viral genetic diversity and geographic distribution change of the variants remain a great challenge to the detection of early HIV infection (Gaudy et al., 2004; Pyne et al., 2013; Xiao et al., 2017). Therefore, higher sensitivity and higher detection capability to various HIV subtypes are essential to the HIV screening assay. The current study included HIV-positive cohorts, with HIV-1 p24 antigen, group O and M, genotypes B/B′, BC, and AE, HIV-2, and low S/Co reactivity (1.0–35.0), to evaluate the sensitivity and detection capability of the LiCA^®^ HIV Ag/Ab assay. LiCA^®^ successfully detected all subtypes of HIV and weak positive samples were recruited. Using the national reference material and WHO international standard for p24 antigen, the LoD of LiCA^®^ was estimated to be 0.73 and 0.90 IU/mL, respectively. These data are favorably comparable to other counterpart assays such as Architect^®^ HIV Ag/Ab combo (LoD = 0.94–1.03 IU/mL), Elecsys^®^ HIV combi PT (LoD = 1.05–1.10 IU/mL), and Centaur^®^ HIV Ag/Ab combo (LoD = 1.89–1.90 IU/mL) (Ly et al., 2012; Muhlbacher et al., 2013).

Furthermore, the high sensitivity of LiCA^®^ is explained with excellent detection of seroconversion panels in comparison to Architect^®^. In 13 panels tested on both assays, LiCA^®^ presented an average of 5.73 (3.42–8.04) days at the first reactive detection after positive RNA and detected more positive samples with a of mean −1.27 (−3.75–1.21) days earlier than Architect^®^.

Paired comparisons in 21,042 clinical patient samples revealed that LiCA^®^ detected HIV Ag/Ab with a slightly better performance in sensitivity (100.00% vs. 99.65%), specificity (99.85% vs. 99.81%), NPV (100.00% vs. 99.99%), and PPV (89.84% vs. 87.85%) than Architect^®^. Excellent assay concordance was observed in nonreactive results (99.85%) but decreased agreement occurred in reactive measurements (88.16%). Most discrepancies (90.00%) primarily resulted from false-positive assays in a low level of S/Co reactivity (<10.00). Previous studies have demonstrated that the Architect^®^ HIV Ag/Ab combo assay yielded a high rate of false-positive results, especially in S/Co values <30.00 (Alonso et al., 2018; Wang et al., 2019). The false reactivity can be generated due to non-specific binding to the immune complex in the Ag–Ab combination assay (Mahajan et al., 2010). The testing discrepancies in the same cohort most likely result from the different Ag/Ab configuration in different assays (Stickle et al., 2002). Notably, there was one subject that was misdetected on Architect^®^ (S/Co = 0.59) but strongly reactive on LiCA^®^ (S/Co = 64.14). This case was from an AIDS inpatient who was at the late stage of ART during our study. The missed detection of Architect^®^ could be explained by the less sensitivity to certain subtypes of HIV (Ly et al., 2012) or the influence on the assay due to viral mutation after treatment (Zuo et al., 2020).

It has been reported that false-positive HIV Ag/Ab screening tests can be caused by other viral infections, autoimmune diseases, and multiple pregnancies (Mahajan et al., 2010; Liu et al., 2016; Adhikari et al., 2018). Our study indicated that no significant cross-reactivity or interference was observed from any of 15 potential interference factors assessed for the LiCA^®^ assay, including auto-antibodies, viral infections such as Epstein–Barr virus and hepatitis viruses, multiple pregnancies such as different stages of normal pregnancy and co-infection pregnancies, and various endogenous interferents. The essence of high specificity and high sensitivity can be attributed to the unique methodology and light-initiated multi-amplification signaling mechanism for the LiCA^®^ assay (Li et al., 2023; Yu et al., 2023).

Combining HIV-1 p24 antigen with antibodies classifies the HIV screening test from the third-generation to the fourth-generation method, which enables the assay to achieve higher sensitivity, reducing the test-negative window to 8–14 days from approximately 3 weeks (Alexander, 2016; Qiu et al., 2017). However, the fourth-generation assay integrates the Ag/Ab reactivity together and can only report a single result in combination of the Ag/Ab S/Co values. In contrast, LiCA^®^ performs immunoassays for detecting HIV p24 antigen and antibodies in two independent cuvettes and separates the S/Co value of p24 antigen from antibodies. Differentiation of the Ag/Ab reactivity can easily identify the preclinical infectious patient with the single-positive p24 antigen (Salmona et al., 2014) and thus facilitate the subsequent confirmatory process for early detection of HIV infection (Muhlbacher et al., 2019; Yang et al., 2022).

In this study, most of the HIV-positive samples were collected from the inpatients with AIDS and the outpatients with highly suspicious history. The positive detection rate was 1.34% (*n* = 21,042). This situation is different from the clinical conditions for screening populations and blood donors, in which the viral prevalence rate can be quite lower (Wang et al., 2019). Another investigation is valuable for further characterization of the assay performance in low prevalence of HIV.

## 5 Conclusion

LiCA^®^ provides a precise and fully automatic platform for measuring HIV-1 p24 antigen and HIV-1/2 antibodies with high sensitivity and specificity. The assay performance is favorably comparable to the well-established Architect^®^ HIV Ag/Ab combo assay in analytical and clinical perspectives. Additionally, LiCA^®^ HIV Ag/Ab can differentiate the reactivity of p24 antigen from antibodies in a separate S/Co result. It is appropriate for use in the clinical routine test for the early detection of HIV.

## Data Availability

The original contributions presented in the study are included in the article/**Supplementary Material**. Further inquiries can be directed to the corresponding authors.
