# Supplementary material for: Evaluation of a new human immunodeficiency virus antigen and antibody test using light-initiated chemiluminescent assay
Source: Front Cell Infect Microbiol. 2025 Jan 31;15:1474127. doi: 10.3389/fcimb.2025.1474127 (PMC11825764; doi:10.3389/fcimb.2025.1474127)
Supplement: Supplementary file 1 [file DataSheet1.docx]

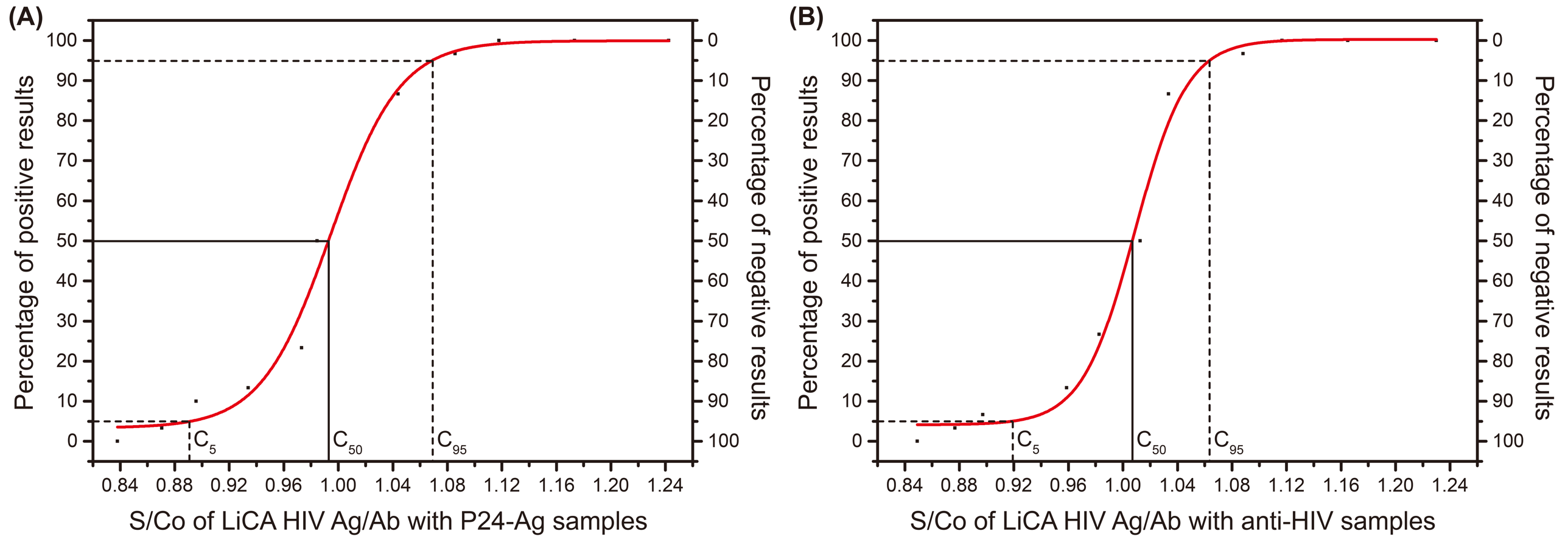


**Supplemental Figure 1:** C_50_ imprecision analysis for the LiCA^®^ HIV Ag/Ab assay with the EP17-A2 protocol. (A) The C_5_~C_95_ intervals were determined to be: (A) -10.20~7.67% away from C_50_ for the p24 antigen; (B) -8.79~5.64% away from C_50_ for the HIV antibodies, respectively.
