# Supplementary material for: Evaluation of a new human immunodeficiency virus antigen and antibody test using light-initiated chemiluminescent assay
Source: Front Cell Infect Microbiol. 2025 Jan 31;15:1474127. doi: 10.3389/fcimb.2025.1474127 (PMC11825764; doi:10.3389/fcimb.2025.1474127)
Supplement: Supplementary file 2 [file Table1.docx]

**Supplemental Table 1:** Study of the detection limit of HIV-1 p24 antigen for the LiCA^®^ HIV Ag/Ab assay. Linear regression equations between the low levels (<10 IU/mL) of p24 antigen concentrations (Y) and assay S/Co ratios (X) were determined to be Y=1.091X–0.365 (R=0.999) with the national reference panel, and Y=2.908X-2.008 (R=0.999) with the WHO international standard, respectively. The detection limits of HIV-1 p24 antigen were then calculated to be 0.73 IU/mL for the national reference panel and 0.90 IU/mL for the WHO international standard, respectively.

| Dilution |  | National reference panel | | |  | WHO^a^ international standard | | |
| --- | --- | --- | --- | --- | --- | --- | --- | --- |
|  |  | IU/mL | S/Co^a^ | Adjudication |  | IU/mL | S/Co^a^ | Adjudication |
| L1 |  | 20.00 | 18.89 | + |  | 1,000.00 | 288.59 | + |
| L2 |  | 10.00 | 9.47 | + |  | 500.00 | 144.91 | + |
| L3 |  | 5.00 | 4.98 | + |  | 250.00 | 72.40 | + |
| L4 |  | 2.50 | 2.64 | + |  | 125.00 | 37.66 | + |
| L5 |  | 1.25 | 1.47 | + |  | 64.00 | 20.35 | + |
| L6 |  | 0.63 | 0.92 | - |  | 32.00 | 10.38 | + |
| L7 |  | 0.31 | 0.59 | - |  | 16.00 | 5.77 | + |
| L8 |  | 0.16 | 0.47 | - |  | 8.00 | 3.43 | + |
| L9 |  | 0.08 | 0.41 | - |  | 4.00 | 2.07 | + |
| L10 |  |  |  | - |  | 2.00 | 1.42 | + |
| L11 |  |  |  |  |  | 1.00 | 1.02 | + |
| L12 |  |  |  |  |  | 0.50 | 0.81 | - |

^a^ WHO, world health organization; S/Co, signal-to-cutoff ratio.

**Supplemental Table 2:** Cross-reactivity study utilized 169 serum samples that were HIV-negative but positive for potential interfering factors such as autoantibodies and other viral infections, detailing the status of these samples.

|  |  |  |  |  |  |  |  |
| --- | --- | --- | --- | --- | --- | --- | --- |
| Category | Source of  interferents | n | Sample matrix | Interferent detection |  | Nonreactive results/Total | |
|  |  |  |  |  |  | LiCA | Architect |
| 1 | Rheumatoid factor | 3 | Serum | Positive |  | 3 | 3 |
| 2 | Auto-antibodies | 10 | Serum | Positive |  | 10 | 10 |
| 3 | Cytomegalovirus (CMV) | 3 | Serum | Positive |  | 3 | 3 |
| 4 | Hepatitis A virus (HAV) | 1 | Serum | Positive |  | 1 | 1 |
| 5 | Hepatitis B virus (HBV) | 54 | Serum | Positive |  | 54 | 54 |
| 6 | Hepatitis C virus (HCV) | 22 | Serum | Positive |  | 22 | 22 |
| 7 | Epstein-Barr virus (EBV) | 2 | Serum | Positive |  | 2 | 2 |
| 8 | Normal pregnancy | 57 | Serum | Positive |  | 57 | 57 |
| 9 | Pregnancy with HBV | 11 | Serum | Positive |  | 11 | 11 |
| 10 | Pregnancy with HCV | 5 | Serum | Positive |  | 5 | 5 |
| 11 | Pregnancy with syphilis | 1 |  |  |  | 1 | 1 |
|  | Total nonreactive results/all tested | | | |  | 169 | 169 |
|  |  | 169 |  |  |  |  |  |
| Remarks: |  |  |  |  |  |  |  |
| 1) All samples were collected from HIV-free patients. | | |  |  |  |  |  |
| 2) Specificity of the LiCA HIV Ag/Ab assay on potentially cross-reacting samples is reported as the number of negative samples for the LiCA HIV Ag/Ab assay on the total number of HIV Ag/Ab negative samples tested. | | | | | | | |

Signal-to-cutoff ratio (S/CO) values for various subtypes of samples positive for different autoantibodies or viral infections.

|  | Architect | LiCA | Classification |  | Architect | LiCA | Classification |
| --- | --- | --- | --- | --- | --- | --- | --- |
| 1 | 0.17 | 0.23 | RF | 85 | 0.09 | 0.18 | HCV |
| 2 | 0.12 | 0.33 | RF | 86 | 0.17 | 0.17 | HCV |
| 3 | 0.16 | 0.19 | RF | 87 | 0.33 | 0.19 | HCV |
| 4 | 0.13 | 0.27 | ANA | 88 | 0.09 | 0.18 | HCV |
| 5 | 0.09 | 0.13 | ANA | 89 | 0.24 | 0.16 | HCV |
| 6 | 0.07 | 0.20 | ANA | 90 | 0.06 | 0.28 | HCV |
| 7 | 0.39 | 0.17 | ANA | 91 | 0.10 | 0.19 | HCV |
| 8 | 0.23 | 0.22 | ANA | 92 | 0.10 | 0.34 | HCV |
| 9 | 0.13 | 0.21 | ANA | 93 | 0.06 | 0.17 | HCV |
| 10 | 0.25 | 0.20 | ANA | 94 | 0.10 | 0.15 | Pregnancy |
| 11 | 0.09 | 0.25 | ANA | 95 | 0.24 | 0.16 | Pregnancy |
| 12 | 0.22 | 0.22 | ANA | 96 | 0.13 | 0.14 | Pregnancy |
| 13 | 0.10 | 0.23 | ANA | 97 | 0.08 | 0.19 | Pregnancy |
| 14 | 0.18 | 0.28 | CMV | 98 | 0.06 | 0.28 | Pregnancy |
| 15 | 0.07 | 0.35 | CMV | 99 | 0.10 | 0.19 | Pregnancy |
| 16 | 0.12 | 0.19 | CMV | 100 | 0.09 | 0.18 | Pregnancy |
| 17 | 0.09 | 0.17 | HAV | 101 | 0.09 | 0.21 | Pregnancy |
| 18 | 0.15 | 0.16 | HBV | 102 | 0.10 | 0.34 | Pregnancy |
| 19 | 0.09 | 0.29 | HBV | 103 | 0.09 | 0.19 | Pregnancy |
| 20 | 0.14 | 0.12 | HBV | 104 | 0.09 | 0.19 | Pregnancy |
| 21 | 0.16 | 0.17 | HBV | 105 | 0.09 | 0.18 | Pregnancy |
| 22 | 0.07 | 0.14 | HBV | 106 | 0.09 | 0.21 | Pregnancy |
| 23 | 0.12 | 0.30 | HBV | 107 | 0.13 | 0.14 | Pregnancy |
| 24 | 0.11 | 0.21 | HBV | 108 | 0.11 | 0.15 | Pregnancy |
| 25 | 0.23 | 0.21 | HBV | 109 | 0.07 | 0.32 | Pregnancy |
| 26 | 0.08 | 0.43 | HBV | 110 | 0.53 | 0.14 | Pregnancy |
| 27 | 0.10 | 0.21 | HBV | 111 | 0.10 | 0.15 | Pregnancy |
| 28 | 0.16 | 0.40 | HBV | 112 | 0.24 | 0.16 | Pregnancy |
| 29 | 0.13 | 0.36 | HBV | 113 | 0.13 | 0.14 | Pregnancy |
| 30 | 0.10 | 0.42 | HBV | 114 | 0.06 | 0.28 | Pregnancy |
| 31 | 0.10 | 0.18 | HBV | 115 | 0.10 | 0.19 | Pregnancy |
| 32 | 0.15 | 0.16 | HBV | 116 | 0.09 | 0.18 | Pregnancy |
| 33 | 0.09 | 0.29 | HBV | 117 | 0.09 | 0.21 | Pregnancy |
| 34 | 0.14 | 0.16 | HBV | 118 | 0.10 | 0.34 | Pregnancy |
| 35 | 0.16 | 0.17 | HBV | 119 | 0.09 | 0.19 | Pregnancy |
| 36 | 0.07 | 0.14 | HBV | 120 | 0.08 | 0.26 | Pregnancy |
| 37 | 0.13 | 0.27 | HBV | 121 | 0.15 | 0.56 | Pregnancy |
| 38 | 0.12 | 0.30 | HBV | 122 | 0.07 | 0.32 | Pregnancy |
| 39 | 0.11 | 0.21 | HBV | 123 | 0.11 | 0.31 | Pregnancy |
| 40 | 0.23 | 0.21 | HBV | 124 | 0.08 | 0.33 | Pregnancy |
| 41 | 0.08 | 0.43 | HBV | 125 | 0.08 | 0.27 | Pregnancy |
| 42 | 0.10 | 0.21 | HBV | 126 | 0.18 | 0.18 | Pregnancy |
| 43 | 0.16 | 0.40 | HBV | 127 | 0.14 | 0.42 | Pregnancy |
| 44 | 0.13 | 0.36 | HBV | 128 | 0.06 | 0.16 | Pregnancy |
| 45 | 0.10 | 0.42 | HBV | 129 | 0.08 | 0.15 | Pregnancy |
| 46 | 0.34 | 0.29 | HBV | 130 | 0.07 | 0.13 | Pregnancy |
| 47 | 0.07 | 0.35 | HBV | 131 | 0.13 | 0.17 | Pregnancy |
| 48 | 0.07 | 0.31 | HBV | 132 | 0.08 | 0.17 | Pregnancy |
| 49 | 0.06 | 0.16 | HBV | 133 | 0.10 | 0.13 | Pregnancy |
| 50 | 0.11 | 0.18 | HBV | 134 | 0.18 | 0.16 | Pregnancy |
| 51 | 0.10 | 0.22 | HBV | 135 | 0.14 | 0.24 | Pregnancy |
| 52 | 0.08 | 0.15 | HBV | 136 | 0.11 | 0.15 | Pregnancy |
| 53 | 0.10 | 0.19 | HBV | 137 | 0.10 | 0.13 | Pregnancy |
| 54 | 0.15 | 0.39 | HBV | 138 | 0.08 | 0.19 | Pregnancy |
| 55 | 0.08 | 0.22 | HBV | 139 | 0.11 | 0.17 | Pregnancy |
| 56 | 0.09 | 0.13 | HBV | 140 | 0.09 | 0.19 | Pregnancy |
| 57 | 0.14 | 0.18 | HBV | 141 | 0.11 | 0.17 | Pregnancy |
| 58 | 0.07 | 0.16 | HBV | 142 | 0.08 | 0.20 | Pregnancy |
| 59 | 0.09 | 0.23 | HBV | 143 | 0.08 | 0.15 | Pregnancy |
| 60 | 0.09 | 0.13 | HBV | 144 | 0.05 | 0.15 | Pregnancy |
| 61 | 0.09 | 0.14 | HBV | 145 | 0.08 | 0.13 | Pregnancy |
| 62 | 0.26 | 0.18 | HBV | 146 | 0.19 | 0.19 | Pregnancy |
| 63 | 0.08 | 0.18 | HBV | 147 | 0.05 | 0.19 | Pregnancy |
| 64 | 0.08 | 0.21 | HBV | 148 | 0.06 | 0.17 | Pregnancy |
| 65 | 0.14 | 0.19 | HBV | 149 | 0.07 | 0.17 | Pregnancy |
| 66 | 0.06 | 0.18 | HBV | 150 | 0.07 | 0.26 | Pregnancy |
| 67 | 0.06 | 0.17 | HBV | 151 | 0.08 | 0.33 | Pregnancy+HBV |
| 68 | 0.15 | 0.18 | HBV | 152 | 0.08 | 0.27 | Pregnancy+HBV |
| 69 | 0.11 | 0.15 | HBV | 153 | 0.06 | 0.16 | Pregnancy+HBV |
| 70 | 0.10 | 0.19 | HBV | 154 | 0.07 | 0.13 | Pregnancy+HBV |
| 71 | 0.30 | 0.18 | HBV | 155 | 0.13 | 0.17 | Pregnancy+HBV |
| 72 | 0.19 | 0.16 | HCV | 156 | 0.14 | 0.24 | Pregnancy+HBV |
| 73 | 0.08 | 0.24 | HCV | 157 | 0.10 | 0.13 | Pregnancy+HBV |
| 74 | 0.06 | 0.21 | HCV | 158 | 0.05 | 0.15 | Pregnancy+HBV |
| 75 | 0.05 | 0.27 | HCV | 159 | 0.08 | 0.13 | Pregnancy+HBV |
| 76 | 0.09 | 0.20 | HCV | 160 | 0.05 | 0.19 | Pregnancy+HBV |
| 77 | 0.10 | 0.20 | HCV | 161 | 0.10 | 0.15 | Pregnancy+HBV |
| 78 | 0.06 | 0.17 | HCV | 162 | 0.24 | 0.16 | Pregnancy+HBV |
| 79 | 0.14 | 0.22 | HCV | 163 | 0.06 | 0.28 | Pregnancy+HBV |
| 80 | 0.17 | 0.17 | HCV | 164 | 0.10 | 0.19 | Pregnancy+HBV |
| 81 | 0.33 | 0.19 | HCV | 165 | 0.10 | 0.34 | Pregnancy+HBV |
| 82 | 0.09 | 0.18 | HCV | 166 | 0.06 | 0.17 | Pregnancy+HBV |
| 83 | 0.17 | 0.17 | HCV | 167 | 0.53 | 0.14 | Pregnancy+ith syphilis |
| 84 | 0.33 | 0.19 | HCV |  |  |  |  |

|  | Architect | mean | min | max | LiCA Mean | min | max |
| --- | --- | --- | --- | --- | --- | --- | --- |
| RF | 3 | 0.15 | 0.12 | 0.17 | 0.25 | 0.19 | 0.33 |
| ANA | 10 | 0.17 | 0.07 | 0.39 | 0.21 | 0.13 | 0.27 |
| CMV | 3 | 0.12 | 0.07 | 0.18 | 0.27 | 0.19 | 0.35 |
| HAV | 1 | 0.09 | 0.09 | 0.09 | 0.17 | 0.17 | 0.17 |
| HBV | 54 | 0.12 | 0.06 | 0.34 | 0.23 | 0.12 | 0.43 |
| HCV | 22 | 0.14 | 0.05 | 0.33 | 0.20 | 0.16 | 0.34 |
| Pregnancy | 57 | 0.11 | 0.05 | 0.53 | 0.21 | 0.13 | 0.56 |
| Pregnancy +HBV | 11 | 0.09 | 0.05 | 0.14 | 0.19 | 0.13 | 0.33 |
| Pregnancy +HCV | 5 | 0.11 | 0.06 | 0.24 | 0.23 | 0.16 | 0.34 |
| Pregnancy + syphilis | 1 | 0.53 | 0.53 | 0.53 | 0.14 | 0.14 | 0.14 |
| Total | 167 | 0.12 | 0.05 | 0.53 | 0.22 | 0.12 | 0.56 |
